# IL-1β and BDNF are associated with improvement in hypersomnia but not insomnia following exercise in major depressive disorder

**DOI:** 10.1038/tp.2015.104

**Published:** 2015-08-04

**Authors:** C D Rethorst, T L Greer, M S P Toups, I Bernstein, T J Carmody, M H Trivedi

**Affiliations:** 1 Center for Depression Research and Clinical Care, Department of Psychiatry, University of Texas Southwestern Medical Center, Dallas, TX, USA; 2 Department of Clinical Sciences, University of Texas Southwestern Medical Center, Dallas, TX, USA

## Abstract

Given the role of sleep in the development and treatment of major depressive disorder (MDD), it is becoming increasingly clear that elucidation of the biological mechanisms underlying sleep disturbances in MDD is crucial to improve treatment outcomes. Sleep disturbances are varied and can present as insomnia and/or hypersomnia. Though research has examined the biological underpinnings of insomnia in MDD, little is known about the role of biomarkers in hypersomnia associated with MDD. This paper examines biomarkers associated with changes in hypersomnia and insomnia and as predictors of improvements in sleep quality following exercise augmentation in persons with MDD. Subjects with non-remitted MDD were randomized to augmentation with one of two doses of aerobic exercise: 16 kilocalories per kilogram of body weight per week (KKW) or 4 KKW for 12 weeks. The four sleep-related items on the clinician-rated Inventory of Depressive Symptomatology (sleep onset insomnia, mid-nocturnal insomnia, early morning insomnia and hypersomnia) assessed self-reported sleep quality. Inflammatory cytokines (tumor necrosis factor-alpha, interleukin (IL)-1β, IL-6) and brain-derived neurotrophic factor (BDNF) were assessed in blood samples collected before and following the 12-week intervention. Reduction in hypersomnia was correlated with reductions in BDNF (*ρ*=0.26, *P*=0.029) and IL-1β (*ρ*=0.37, *P*=0.002). Changes in these biomarkers were not associated with changes in insomnia; however, lower baseline levels of IL-1β were predictive of greater improvements in insomnia (F=3.87, *P*=0.050). In conclusion, improvement in hypersomnia is related to reductions in inflammatory markers and BDNF in persons with non-remitted MDD. Distinct biological mechanisms may explain reductions in insomnia.

## Introduction

Sleep has a significant role in the development treatment of major depressive disorder (MDD). Poor sleep quality is a common symptom of MDD and is one of the most prevalent residual symptoms following antidepressant treatment.^1, 2, 3^ Importantly, these residual sleep disturbances are predictive of relapse in following MDD remission.^4, 5^ As a result, understanding the biological mechanisms related to changes in sleep are important steps in moving toward optimal treatment of MDD.

Evidence suggests a biological link between sleep and depression. Certain biomarkers implicated in the development of MDD and treatment response have also been linked to sleep quality. For example, low levels of brain-derived neurotrophic factor (BDNF) are observed in persons with MDD,^6^ and many treatments for MDD result in increased BDNF.^7, 8^ Increases in BDNF have also been associated with increased non-rapid eye movement (non-REM) sleep and slow wave activity during sleep.^9^ Similarly, elevations in pro-inflammatory cytokines, particularly interleukin (IL)-6, IL-1β and tumor necrosis factor-alpha (TNF-α), have been implicated in the development and treatment of MDD.^10^ IL-1β and TNF-α are generally thought to enhance sleep; however, extreme elevations in IL-1β and TNF-α can impair sleep.^11, 12^

Sleep disturbances can present as either insomnia or hypersomnia in MDD, with hypersomnia as a defining symptom of atypical depression. Distinguishing between atypical and melancholic depression has important clinical relevance as differential treatment responses have been observed in patients with atypical features.^13, 14, 15^ However, previous research of these biological correlates of sleep disturbances is limited in that it does not distinguish between insomnia and hypersomnia. Identification of biomarkers that uniquely predict or correlate with improvements in hypersomnia and insomnia is an important step toward more effective treatment of MDD.

Exercise has proven efficacious as a monotherapy as well as augmentation treatment for MDD.^16, 17, 18, 19, 20^ BDNF and inflammatory cytokines have been implicated in the antidepressant effects of exercise.^21, 22, 23^ Furthermore, exercise has been shown to reduce insomnia independent of improvement in depressive symptoms.^24^ The purpose of this paper is to identify biological correlates and predictors of improvements in self-reported hypersomnia and insomnia through a secondary analysis of the Treatment with Exercise Augmentation for Depression (TREAD) trial. We hypothesize the following: (1) increases in BDNF but decreases in pro-inflammatory cytokines will be associated with improvements in self-reported insomnia and hypersomnia and (2) baseline levels of these biomarkers will predict improvements in self-reported insomnia and hypersomnia.

## Materials and methods

The TREAD trial was a randomized trial comparing two doses of aerobic exercise as augmentation treatment for non-remitted MDD. Full study methodology has been previously published;^20, 25^ provided below is a brief description of study procedures relevant to the current analysis. The study protocol was approved by the local institutional review board and all the subjects signed institutional review board-approved informed consent documents before engaging in any study procedures.

### Subjects

In all, 126 eligible subjects were enrolled and randomized to one of the two treatment arms. To be eligible, individuals must have been in the age range 18–70 and had a diagnosis of a non-remitted MDD, based on the Structure Clinical Interview for DSM-IV Axis I Disorders. Non-remission was defined as a score of ⩾14 on the Hamilton Rating Scale for Depression following 2 to 6 months of treatment with a selective serotonin reuptake inhibitor (SSRI), with at least 6 weeks at an adequate dose.

### Exercise intervention

Subjects were randomly allocated to one of the two exercise groups. In the 12-week intervention, one group was prescribed 4 kilocalories per kilogram of bodyweight per week (KKW) and the other group was prescribed 16 KKW. The 16 KKW dose was designed to be equivalent to the current physical activity recommendation of 150 min per week of moderate intensity exercise.^26^ Exercise intensity was self-selected during all the sessions and monitored with a Polar 610i heart rate monitor. In week 1, both the groups completed the entire exercise dose during the sessions supervised by trained personnel at The Cooper Institute. In week 2, two supervised sessions were conducted with the remaining exercise dose completed during home-based exercise sessions. In each subsequent week, the exercise dose was completed in one supervised exercise session per week with the remaining dose completed during the home-based sessions.

### Clinical assessments

The clinician-rated Inventory of Depressive Symptomatology (IDS-C)^27^ was used to assess depressive symptoms. The four sleep-related items on the IDS-C (sleep onset insomnia, mid-nocturnal insomnia, early morning insomnia and hypersomnia) were used to assess self-reported sleep quality. Each sleep item was scored on a scale of 0–3, with higher scores indicating greater symptom severity. A total insomnia score (range 0–9) was created by summing the first three insomnia-related items (sleep onset insomnia, mid-nocturnal insomnia and early morning insomnia). Validity of the IDS sleep items has been established through comparison with sleep diaries.^28^ Blinded raters completed all assessments at baseline and at the 12 weekly visits.

### Biomarker collection and analysis

Of the 126 randomized TREAD subjects, 108 signed additional consent for blood analysis at baseline (105 baseline samples were available). Seventy-three of these completed the study and provided week 12 samples. Those agreeing to provide blood samples and those who refused did not differ on any demographic or baseline characteristic and those providing a sample at week 12 did not differ from those who did not. All the samples were drawn in the morning; subjects fasted a minimum of 3 h before blood draw and were at least 24 h from the last exercise session. A total 10 ml of peripheral venous blood was drawn and centrifuged at 900 r.p.m. for 10 min at room temperature to separate the blood components. Serum was subsequently frozen at 80 °C until the time of analysis. We analyzed samples in duplicate using a multiplexed chemoilluminescent ELISA (enzyme-linked immunosorbent assay) method (MesoScale Discovery, Gaithersburg, MD, USA) for IL-1β, IL-6 and TNF-α. The plates were read using the MSD Discovery Workbench analyzer and software package (MesoScale Discovery). All the data were calibrated using standard curves generated for each cytokine. For the analysis of serum BDNF, samples were analyzed in triplicate according to the manufacturer's protocol using R&D Human BDNF Quantikine kits (R&D Systems, Minneapolis, MN, USA).

### Statistical analysis

Due to the non-normality of the biomarker and sleep variables, Spearman's non-parametric rank-order correlation coefficient was used to examine the relationship between change in each biomarker and change in hypersomnia and insomnia. Change variables (week 12—baseline) were only calculated for subjects who provided week 12 data to eliminate time as a confounding factor.

A linear mixed model repeated-measures analysis examined the relationship between each baseline cytokine level and hypersomnia and insomnia over the 12-week study period. Each model contained fixed effects terms for baseline biomarker level (pg ml^−1^), time and biomarker level by time interaction. Group, group by time and group by cytokine interactions were tested in the model but removed as they were all nonsignificant. The intercept was included as a random effect. Time was log transformed to provide a more linear relationship with outcome. Restricted maximum-likelihood estimation and type 3 tests of fixed effects were used, with the Kenward–Roger correction applied to the spatial power covariance structure. Covariates were selected on the basis of previous analysis of the TREAD data (Trivedi 2011). Covariates included in the model were baseline insomnia or hypersomnia, IDS-C score minus sleep items, family history of MDD, recurrent MDD, age, sex, race, body mass index, Short Form Health Survey (SF-36) mental subscale and SF-36 physical subscale. Covariates and baseline biomarker level were centered. Examination of the normality of the residuals in this analysis indicate that the use of non-parametric techniques was not necessary. All the analyses were carried out using SAS software, version 9.2 (SAS Institute, Cary, NC, USA, code available upon request).

## Results

Demographic and baseline clinical characteristics of the study sample are presented in Table 1. Means and standard deviations are presented for all variables. Due to non-normality, medians and interquartile ranges are also reported for the sleep variables and biomarkers. One baseline value for TNF-α was 5 s.d. above the sample mean and thus excluded from further analysis. Two subjects had missing baseline values for IL-1β and were also excluded. An additional subject had no value for 12-week IL-1β—this subject was included in the mixed model but excluded from the correlation analysis.

### Relationship between change in biomarkers and change in sleep quality

Table 2 presents the mean change in depressive symptoms (IDS-C total score), IDS-C total insomnia, IDS-C hypersomnia and each biomarker. Spearman correlation coefficients between the change in each biomarker and changes in insomnia and hypersomnia are presented in Table 3. In summary, there was a significant correlation between change in IL-1β and change in hypersomnia (*ρ*=−0.37, *P*=0.003) and between change in BDNF and change in hypersomnia (*ρ*=0.26, *P*=0.029). No other correlations were significant.

### Baseline biomarkers as predictors of change in sleep quality

**Figure 1:**
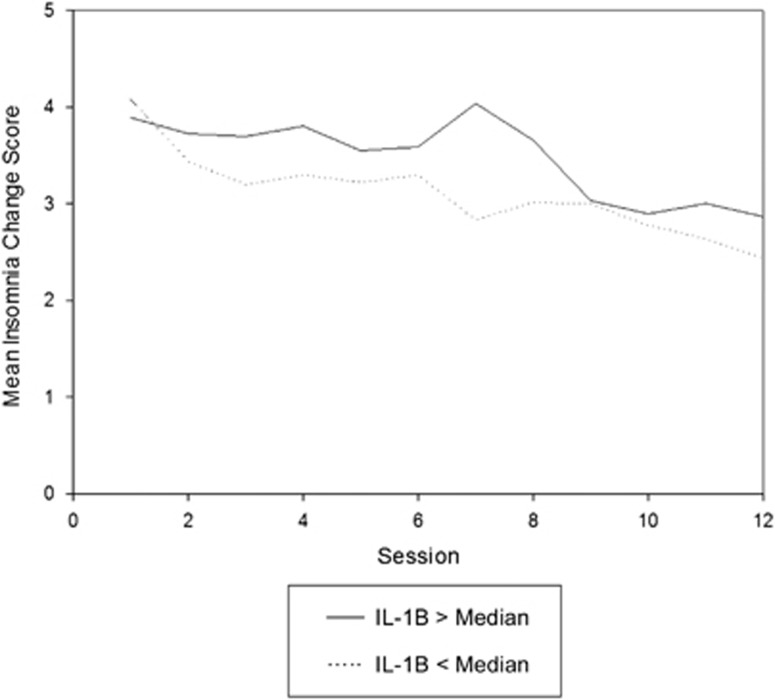
Changes in mean insomnia score by baseline IL-1B level. IL, interleukin.

Mixed model analyses revealed a significant IL-1β × time interaction (F=3.87, *P*=0.0498). This interaction is illustrated in Figure 1, which depicts the least squares means for insomnia by week for the sample following a median split of baseline IL-1β. The analysis and figure indicate that for those with lower levels of IL-1β at baseline exercise resulted in lower insomnia scores throughout the 12-week study. No other baseline biomarker was a significant predictor in change of sleep quality (Table 4).

## Discussion

The results of our analysis indicate a relationship between changes in inflammatory and neurotrophic biomarkers and changes in hypersomnia in a study of exercise augmentation for non-remitted MDD. Specifically, reductions in BDNF and IL-1β are related to reductions in hypersomnia. Furthermore, low baseline levels of IL-1β were predictive of greater reductions in insomnia during the 12-week trial. These findings provide insight into the relationship between exercise and the nature of sleep impairment in patients with MDD. Given the role of sleep in the development, treatment and recurrence of MDD,^1, 4, 29, 30^ our findings also have potential implications in the treatment of MDD.

Previous research has demonstrated a positive effect of exercise on sleep quality;^31, 32, 33^ however, the mechanisms underlying this effect have not been thoroughly examined. Santos *et al.*^34^ have proposed the anti-inflammatory effect of exercise as a mechanism for improved sleep and previous studies have supported this hypothesis.^35, 36^ The current analysis is the first to examine this relationship in subjects with MDD. The relationship of reduced IL-1β with reduced hypersomnia fits with previous research. Though IL-1β is generally thought to enhance sleep, extreme increases in inflammation appear to have detrimental effects on sleep quality.^11, 12^ In animals, injections of IL-1β and TNF-α result in increased non-REM sleep time and slow wave activity during the non-REM sleep.^37^ In humans, increases in inflammation following the administration of interferon-alpha in hepatitis C patients is predictive of decreases in self-reported sleep quality assessed by the Pittsburgh Sleep Quality Index.^38^ Lower self-reported Pittsburgh Sleep Quality Index scores are also associated with an increased inflammatory response to stress.^39^ As a whole, this suggests a negative feedback loop in which sleep, inflammation and depression interact and progressively worsen. The results of the current analysis, along with our previous findings in this sample,^21, 24^ suggest that exercise may be resetting this negative feedback loop.

Our results also implicate BDNF in improvements in sleep quality following exercise, as decreases in BDNF were associated with decreased hypersomnia. This finding is in contrast to our initial hypothesis that improvements in sleep would be related to increases in BDNF. Our intial hypothesis was based on previous research that demonstrated BDNF-dependent changes in sleep quality. In animals, intracerebroventrical injections of BDNF increase both non-REM and REM sleep^40^ and increased slow wave activity during non-REM sleep.^9^ In humans, non-REM sleep slow wave activity was higher during recovery sleep in Val/Val genotype compared with Val/Met genotype.^41^ Finally, ketamine treatment of MDD has been found to result in associated increases in BDNF and non-REM slow wave activity.^42^ The discrepancy between our results and our initial hypothesis may be owing to the unique biological underpinnings of hypersomnia and help further highlight the possible differential biomarker associations between hypersomnia and insomnia.

Hypersomnia is a symptom most commonly associated with atypical depression. Atypical depression is characterized by mood reactivity, meaning that the individual will experience an improvement in mood in response to positive events. Other symptoms of atypical depression include increased appetite and/or weight gain, leaden paralysis and interpersonal sensitivity. Atypical depression may be a result of reduced function in the hypothalamic–pituitary–adrenal (HPA) axis, in contrast to melancholic depression, which has been linked with overactivity of the HPA axis.^43, 44^ This overactivity of the HPA axis in melancholic depression has been linked to reductions in BDNF expression.^45^ As a result, normalization of symptoms in atypical depression would result in increased HPA activity and decreased BDNF production. In this scenario, decreased BDNF production would be associated with decreases in hypersomnia, which matches our findings.

There was no relationship between changes in these inflammatory markers or BDNF with changes in insomnia. Previous analysis of the TREAD data demonstrated significant reductions in insomnia symptoms;^24^ when combined with the current findings, this suggests that other biological mechanisms are likely responsible for exercise-induced improvements in insomnia in this sample of persons with non-remitted MDD. Potential mechanisms not examined in our current study include hypothalamic–pituitary axis and metabolic function. Dysregulation of the hypothalamic–pituitary axis has been related to insomnia,^46, 47, 48^ while exercise training improves HPA function.^49, 50^ Similarly, impaired glucose tolerance in patients with type 2 diabetes is associated with poorer sleep quality,^51^ whereas exercise improves glucose tolerance.^52, 53^

The associations of inflammatory and neurotrophic markers with an improvement in hypersomnia but not insomnia provide further support for the differing biological underpinnings of atypical depression. Previous research has identified differences in inflammation, HPA-axis function and metabolic indices in patients with atypical depression.^54, 55, 56, 57^ It is likely that these biological differences underlie the differential treatment response observed in patients with atypical features.^13, 14, 15^

One weakness of the current analysis is that subjects in the TREAD trial were taking SSRIs for at least 2 months before initiation of the exercise intervention. SSRIs can also alter levels of inflammatory markers and BDNF in patients with depression, so it is difficult to conclude whether our findings would generalize to treatment-naive patients. Furthermore, though SSRIs impact both inflammatory markers and BDNF,^58, 59, 60^ insomnia is a common residual symptom following SSRI treatment^1, 2^ and SSRIs themselves can cause disrupted sleep.^61^ Therefore, it is possible that SSRI effects may confound the analysis of the relationship between sleep and the assessed biomarkers.

Previous research supports augmentation strategies targeting sleep symptoms to improve MDD treatment outcomes.^29, 30^ Optimal implementation of exercise in the treatment of MDD and sleep disturbances will require an understanding of the underlying biological mechanisms. The results of the current analysis implicate pro-inflammatory cytokines and BDNF in exercise-induced improvements in sleep quality but also suggest that other biological mechanisms are likely involved. The fact that changes in IL-1β and BDNF were only related to changes in hypersomnia further support the need to identify biological markers that differentiate across different symptom profiles. These findings highlight the need for further research examining the biological mechanisms linking exercise and sleep. Given the importance of treating sleep in improving treatment outcomes, future work aimed at understanding these biological mechanisms in MDD is especially important.

## Figures and Tables

**Table 1:** Baseline demographic and clinical characteristics

*Variable*	N	*Mean (s.d.)*	*Median (IQR)*
Age (years)	105	47.51 (9.44)	
Female (%)	105	80	
IDS-C	105	34.14 (7.25)	
IDS insomnia	105	4.25 (2.18)	4.00 (3.00–6.00)
IDS hypersomnia	105	0.67 (0.90)	0.00 (0.00–1.00)
BDNF	104	19.06 (6.23)	18.04 (14.96–22.91)
IL-1β	103	0.10 (0.07)	0.08 (0.06–0.13)
IL-6	105	0.80 (0.68)	0.73 (0.47–1.08)
TNF-α	104	6.35 (6.42)	5.50 (4.33–7.02)
BMI	105	30.89 (6.31)	
SF-36 physical health	101	80.18 (20.80)	
Recurrent MDD (%)	105	69.52	
Family history of MDD (%)	105	67.62	
Length of illness (years)	104	19.96 (12.29)	
Length of current MDD episode (years)	105	7.10 (8.29)	

Abbreviations: BDNF, brain-derived neurotrophic factor; BMI, body mass index; IDS-C, Inventory of Depressive Symptoms—clinician rated; IL, interleukin; IQR, interquartile range; MDD, major depressive disorder; TNF-α, tumor necrosis factor-alpha.

**Table 2:** Mean change in depression, sleep and biomarkers

*Variable*	N	*Mean (s.d.)*	t	P*-value*
IDS-C	98	−11.42 (10.53)	−10.74	<0.001
IDS-C insomnia	99	−1.81 (2.32)	−7.76	<0.0001
IDS-C hypersomina	99	−0.25 (0.94)	−2.67	0.009
BDNF	69	0.11 (3.52)	0.26	0.793
IL-1β	68	0.03 (0.29)	0.97	0.333
IL-6	71	−0.13 (0.80)	−1.34	0.184
TNF-α	71	−0.17 (0.98)	−1.43	0.157

Abbreviations: BDNF, brain-derived neurotrophic factor; IDS-C, Inventory of Depressive Symptoms—clinician rated; IL, interleukin; TNF-α, tumor necrosis factor-alpha.

**Table 3:** Spearman correlation coefficients (rs) between the change in each inflammatory cytokine level and change in insomnia and hypersomnia

	*BDNF*			*IL-6*			*TNF-α*			*IL-1β*		
	n	ρ	P*-value*	n	ρ	P*-value*	n	ρ	P*-value*	n	ρ	P*-value*
Insomnia	68	−0.09	0.481	70	0.05	0.642	70	−0.04	0.731	67	−0.03	0.802
Hypersomnia	68	0.26	**0.029**	70	0.05	0.674	70	0.08	0.511	67	0.37	**0.002**

Abbreviations: BDNF, brain-derived neurotrophic factor; IL, interleukin; TNF-α, tumor necrosis factor-alpha.

Bold texts highlight *P*-values <0.05.

**Table 4:** Time × baseline biomarker interaction test of fixed effects from mixed model analysis

	*BDNF* × *time*			*IL-6* × *time*			*IL-1β* × *time*			*TNF-α* × *time*		
	n	*F*	P*-value*	n	*F*	P*-value*	n	*F*	P*-value*	n	*F*	P*-value*
Insomnia	99	2.11	0.148	100	2.36	0.125	98	3.87	**0.050**	100	0.21	0.648
Hypersomnia	99	0.00	0.958	100	0.00	0.954	98	0.38	0.536	100	0.03	0.854

Abbreviations: BDNF, brain-derived neurotrophic factor; IL, interleukin; TNF-α, tumor necrosis factor-alpha.

Bold texts highlight *P*-values <0.05.

## Notes

TLG has received consulting fees from H Lundbeck A/S and Takeda Pharmaceuticals International. IB has received support from the Joint Research Committee of the National Council Of State Boards Of Nursing, book royalties from Sage Publications, owns stock in Merck, Bristol-Myers Squibb and DuPont, EI. de Nemours & CC. MHT is or has been an advisor/consultant to Abbott Laboratories, Abdi Ibrahim, Akzo (Organon Pharmaceuticals), Alkermes, AstraZeneca, Axon Advisors, Bristol-Myers Squibb Company, Cephalon, Cerecor, Concert Pharmaceuticals, Eli Lilly and Company, Evotec, Fabre Kramer Pharmaceuticals, Forest Pharmaceuticals, GlaxoSmithKline, Janssen Global Services, LLC, Janssen Pharmaceutica Products, LP, Johnson & Johnson PRD, Libby, Lundbeck, Meade Johnson, MedAvante, Medscape, Medtronic, Merck, Mitsubishi Tanabe Pharma Development America, Naurex, Neuronetics, Otsuka Pharmaceuticals, Pamlab, Parke-Davis Pharmaceuticals, Pfizer, PgxHealth, Phoenix Marketing Solutions, Rexahn Pharmaceuticals, Ridge Diagnostics, Roche Products, Sepracor, SHIRE Development, Sierra, SK Life and Science, Sunovion, Takeda, Tal Medical/Puretech Venture, Targacept, Transcept, VantagePoint, Vivus and Wyeth-Ayerst Laboratories. In addition, he has received research support from Agency for Healthcare Research and Quality (AHRQ), Corcept Therapeutics, Cyberonics, National Alliance for Research in Schizophrenia and Depression, National Institute of Mental Health, National Institute on Drug Abuse, Novartis, Pharmacia & Upjohn, Predix Pharmaceuticals (Epix) and Solvay Pharmaceuticals. The remaining authors declare no conflict of interest.
